# Cardiovascular Disease Risk in Obstructive Sleep apnea: An Update

**DOI:** 10.4172/2167-0277.1000283

**Published:** 2018-02-12

**Authors:** Mena Yacoub, Irini Youssef, Moro O Salifu, Samy I McFarlane

**Affiliations:** 1 Department of Cardiology, Northside Hospital at Tampa Bay Heart Institute, St. Petersburg, Florida, USA; 2 Department of Medicine, State University of New York Downstate Medical Center, Brooklyn, New York, USA

## Introduction

**Figure 1:**
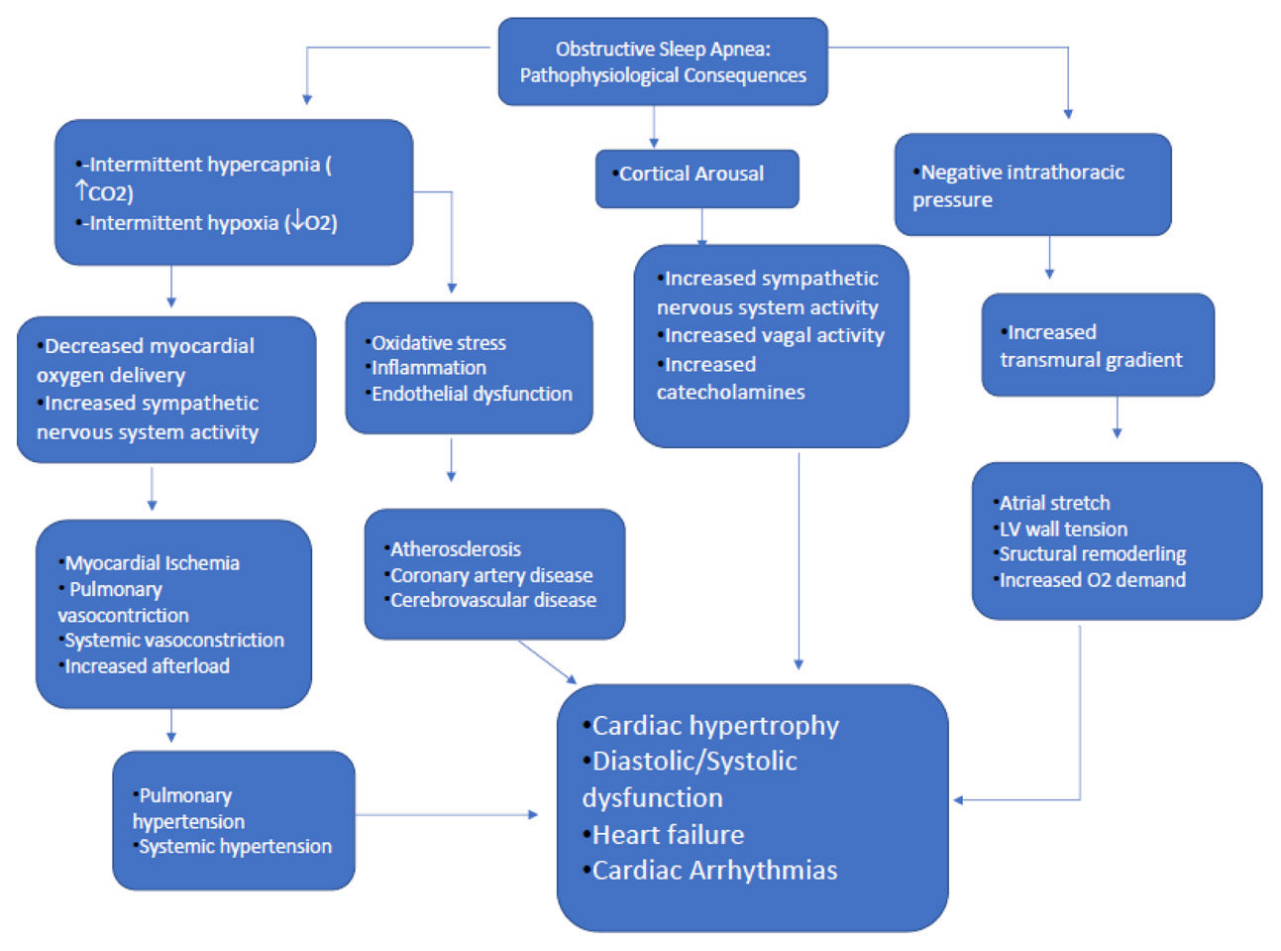
Pathophysiological mechanisms linking obstructive sleep apnea to cardiovascular disease.

**Figure 2:**
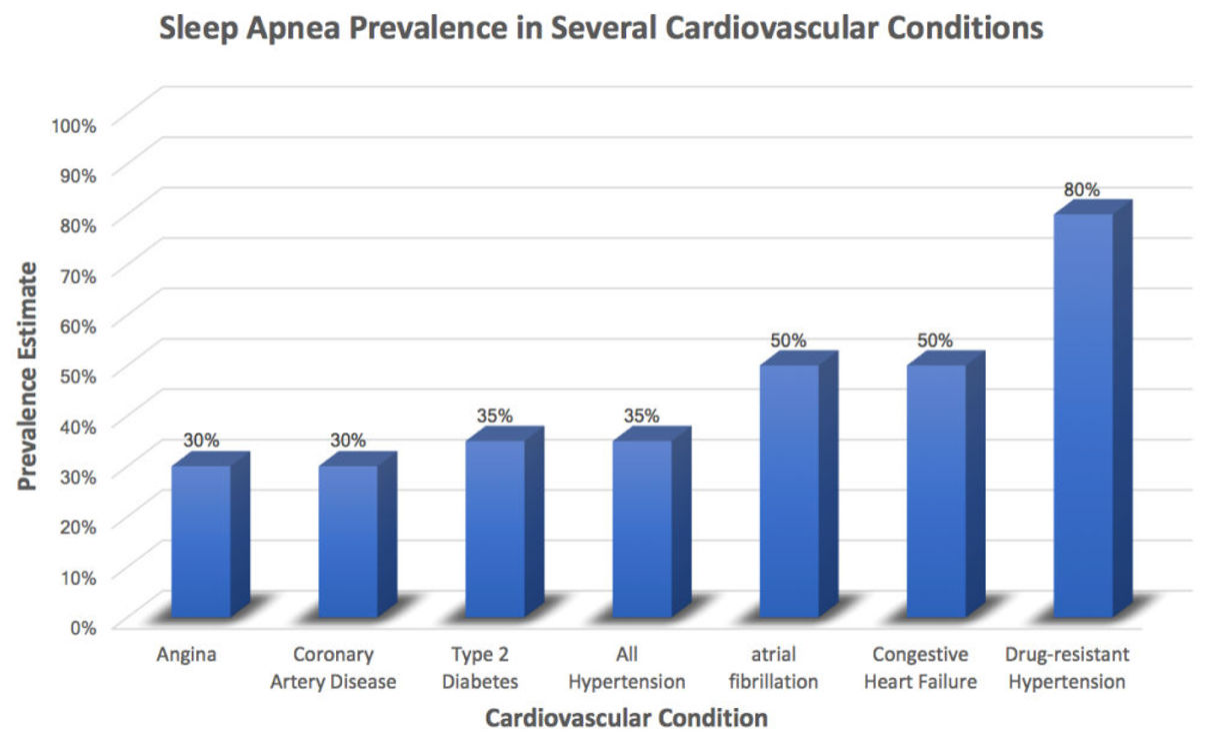
Prevalence estimates of sleep apnea in several cardiovascular conditions.

Cardiovascular disease (CVD) is the number one cause of death globally, accounting for 31% of all deaths in the world, and one out of every three deaths in the United States, in 2017 [1]. It is estimated that 90% of CVD is preventable, with traditional risk factors including tobacco use, excessive alcohol consumption, and unhealthy diet and physical inactivity, leading to hypertension, diabetes, dyslipidemia, and obesity. The epidemic of obesity has increased over the past decade, affecting an estimated 37.7% of adults, and 18% of children, in the US, and projected to rise to 51% of the population, a 130% increase, by 2030 [2]. This obesity epidemic has ushered in a new, highly prevalent, but largely underdiagnosed CVD risk factor: obstructive sleep apnea (OSA). OSA affects 34% of men, and 17% of women in the United States [3]. It is characterized by repetitive episodes of hypoventilation and complete apnea during sleep caused by total pharyngeal collapse and airway obstruction, despite normal breathing effort and drive. OSA’s impact on CVD appears to be due to recurrent cardio metabolic perturbations experienced when repetitively attempting to breath against an occluded airway, precipitating nightly episodes of hypoxia, sleep disturbance, and sympathetic nervous system surges, culminating in elevated blood pressure and heart rate, endothelial dysfunction, systemic inflammation and insulin resistance-all mechanisms involved in the pathogenesis of CVD (Figure 1) [4]. Accumulating evidence has linked OSA to multiple cardiovascular disorders including hypertension, type 2 DM, coronary artery disease, heart failure and cardiac arrhythmias (Figure 2).

## OSA and HTN

OSA is a well-recognized, if not the most common, cause of secondary hypertension [5]. A prospective study by Peppard et al. on 709 patients with OSA found a dose response association between apnea-hypopnea episodes and the presence of hypertension, with patients with more than 15 apnea-hypopnea episodes a night having an odds-ratio of 2.89 for the presence of hypertension [6]. Another study by Logan et al. on patients with drug resistant hypertension (defined as a BP of > =140/90 in patients taking a combination of three or more anti-hypertensives, titrated to maximal dosage) reported a prevalence of OSA of 83% [7].

Several mechanisms explaining the pathophysiology effects of OSA on the development of HTN have been proposed. They include reflex surges in sympathetic nervous system activity with consequent increases in catecholamine levels and elevations in BP caused by repetitive hypoxemic and hypercapnic episodes, and hypoxemic stress induced release of vasoactive and trophic substances that trigger vasoconstriction, even persisting into the daytime [8].

Effective treatment of OSA has shown promising results in the treatment of hypertension. In a study of 1889 patients with OSA, without HTN, by Marin et al, the authors reported a five-times greater risk of new onset HTN in patients with OSA untreated by CPAP compared to patients treated with CPAP, after adjusting for AHI, age and sex [9]. The impact of CPAP treatment on HTN progression was also reported by Drager and colleagues. In their study of 36 male patients with severe OSA and prehypertension, patients randomized to the CPAP arm showed significant reductions, up to 8 mmHg changes, in both systolic and diastolic blood pressure, and a 39% significant reduction in the frequency of prehypertension, compared to controls. This cumulative data on hypertension has led to efforts by the Joint National Committee on Prevention, Detection, Evaluation, and Treatment of High Blood Pressure to identify OSA as a preventable cause of HTN [10].

## OSA and Diabetes Mellitus

OSA is also independently associated with type 2 diabetes. Several prospective studies have examined the incidence of type 2 DM in patients with OSA. In their study of 1387 patients, Reichmuth et al. found an odd of diabetes four times greater in patients with OSA compared to control [11]. Another study by Marshall et al. found an OR of 13.45 of diabetes in their OSA population, even after adjusting for age, sex, BMI, waist, BP, and HDL [12]. The incidence of diabetes is also independently influenced by the severity of oxygen desaturations [13]. Moreover, glycemic control is adversely affected by OSA severity, as patients with severe OSA have been found to have higher A1c levels compared to patients with mild to moderate disease [14].

The mechanisms involved in the development of insulin resistance and diabetes in patients with OSA have been extensively studied and described. These include hypoxia induced sympathetic stimulation causing increased hepatic gluconeogenesis and decreased skeletal muscle uptake of glucose, resulting in hyperglycemia; and metabolic dysfunction caused by sympathetic nervous system activation, oxidative stress, and systemic inflammation, all of which cause decreased insulin sensitivity and impair glucose metabolism [15].

The impact of CPAP treatment of OSA on improving glucose homeostasis has shown results ranging from improvements in post-prandial glucose levels to modest reductions in A1c. In their study of 25 patients with type 2 DM, Babu et al. reported significant reductions in hemoglobin A1c levels with CPAP use, as well as greater reductions in patients with longer duration of CPAP use [16]. CPAP also proves valuable in significantly improving insulin sensitivity, as shown by Martinez-Ceron et al. in their randomized control trial of 50 patients with OSA treated with CPAP.

## OSA and CAD

OSA is a significant underlying risk factor for CAD. The presence and severity of OSA has been found to be independently associated with the extent of coronary artery calcification, a marker often used as a surrogate for coronary artery disease [17]. Data from over 6000 patients in the Sleep Heart Healthy Study also revealed an independent association between OSA and the incidence of CAD, especially in patients with severe OSA [18]. Another observational study of 1436 patients, by Shah et al. found a significant association between OSA and coronary artery events and cardiovascular death, after adjusting for traditional CVD risk factors, including HTN and obesity [19]. Also compared to controls, OSA is associated with a higher rate of worse outcomes in patients presenting with ACS, including higher incidence of cardiac death and re-infarction [20].

The mechanisms leading to atherosclerosis in OSA are related to the repeated hypoxic episodes which induce oxidative stress, vascular damage, systemic inflammation, and endothelial dysfunction. Loss of endothelium-derived nitric oxide production and subsequent impairment of vascular relaxation has been reported in patients with OSA [21]. Several inflammatory pathways, all induced by chronic hypoxemia, have also been described in patients with OSA, leading to increased serum levels of inflammatory substances and adhesion molecules which facilitate the atherosclerotic process [22]. Several studies have also elucidated the role of OSA in increasing platelet activity and reducing fibrinolysis; outcomes responsible for the final pathogenic process of acute coronary syndromes [23].

The utility of CPAP treatment in the reduction of CAD and ACS has been studied; however, definitive results have not been well characterized. The RICCADSA (Randomized Intervention with Continuous positive airway pressure in Coronary Artery Disease and OSA) trial of 244 patients with OSA and angiographically proven CAD found a significant cardiovascular risk reduction in patients who used CPAP for more than 4 hours per night, although it did not find a significant difference in endpoints including myocardial infarction, stroke, or cardiovascular mortality [24]. Another trial by McEvoy on 2687 patients with OSA and CAD did not find a statistically significant reduction in those end-points in patients treated with CPAP [25]. The ongoing trial ISAACC (Continuous Positive Airway Pressure in Patients with Acute Coronary Syndrome and Obstructive Sleep Apnea) trial, a trial which will include more than 1800 patients with recent ACS and OSA, is currently underway and will assess the impact of CPAP treatment on the outcome of major cardiovascular events.

## OSA and CHF

OSA continuously exposes the cardiovascular system to several derangements secondary to intermittent episodes of hypoxia, causing increased oxidative stress, systemic inflammation, exaggerated fluctuations in intrathoracic pressure and sympathetic hyper-stimulation. The combination of these outcomes ultimately causes increased strain on the myocardium, leading to impairment in myocardial contractility and the development and progression of heart failure. Approximately 35% of patients with heart failure with preserved ejection fraction, and 30% of patients with HF with reduced ejection fraction, have OSA [8]. The Sleep Heart Health Study on 709 patients reported that OSA was independently associated with a 2.38 odds of having HF compared to controls [18]. Furthermore, in patients with existing heart failure, the presence of untreated OSA has been associated with significantly greater death rates [26].

OSA appears to contribute to heart failure both in the initial process, and in the perpetuation of the disease. Nocturnal oxygen desaturation leads to sympathetic system activation and subsequent systemic and pulmonary vasoconstriction, causing elevations in blood pressure. These alterations in afterload on both the left and right ventricles lead to ventricular hypertrophy; the most important predisposing factor for cardiac failure. Thereafter, OSA contributes to progression of heart failure in those biologically susceptible on a nightly basis by eliciting greater sympathetic outflow to the heart and continuous elevations in afterload. Moreover, repetitive exposure to large negative intrathoracic pressures produces an increased load burden on the myocardium, truly unique to OSA. These abrupt increases in the transmural pressures and afterload cause profound reductions in stroke volume, and greater reflex surges in central sympathetic outflow, ultimately exacerbating cardiac failure [8].

In patients with HF, treatment of OSA has shown improvement in symptoms. In the first randomized control study of 24 patients with severe systolic heart failure and OSA, the use of CPAP for one month showed a 9% improvement in left ventricular ejection fraction, compared to usual medical care, and reduced daytime systolic BP and HR [27]. Mansfield and Colleagues have reported results of a similar study, in which patients randomized to 3 months of CPAP versus usual care, had a 5% improvement in left ventricular ejection fracture compared to control, lower nighttime urinary norepinephrine levels, and more improved quality of life [28].

## OSA and Cardiac arrhythmias

Cardiac arrhythmias occur more frequently in patients with OSA, and are directly related to the number of hypoxic episodes. Nocturnal arrhythmias, including ventricular tachycardia, sinus arrest, premature ventricular contractions, and atrial fibrillation, are reported more often in patients with sleep disordered breathing. In a study by Guilleminault et al. on 400 patients with OSA, the authors reported a 48% prevalence of cardiac arrhythmias. Notably, all of the patients (n=50) who had tracheostomy did not have any arrhythmias post-operatively [29]. A subgroup analysis of patients from the Sleep Heart Health Study revealed up to a fourfold increase in the prevalence of cardiac arrhythmias in patients with severe OSA compared to control. After adjusting for age, sex, and BMI, patients with OSA had an OR of 4.02 for AF, 3.4 for ventricular tachycardia, and 1.74 for ventricular ectopy [30]. In our meta-analysis by Youssef et al. on 19, 837 patients from prospective studies reporting on the incidence of atrial fibrillation in patients with OSA, we found a twofold increased risk of development of atrial fibrillation in patients with OSA, compared to controls.

There are several proposed mechanisms by which arrhythmias could occur in OSA. Repeated oxygen desaturations during obstructive episodes induce baroreflex and chemo reflex activation. This persistent exposure has been shown to induce abnormal electrical remodeling of the myocardium, most commonly the atrium, a potential explanation for the high prevalence of atrial fibrillation in OSA. The dramatic elevations in atrial transmural pressures have also been found to trigger stretch activated atrial ion channels. Enhanced sympathetic activity may also be a direct cause of triggered automaticity causing ectopic arrhythmias. On the other hand, prolonged apneic-hypopneic episodes are known to elicit cardiac reflex vagal stimulation, ultimately ending in bradyarrhthmias.

The effect of CPAP therapy on OSA appears to mitigate the occurrence of arrhythmias. Kanagala et al. found that patients with OSA and AF who did not receive CPAP treatment after cardio version were two times more likely to relapse [31]. In another study, Patel et al. evaluated 3000 patients undergoing catheter ablation of AF and found an 11% significant reduction in recurrence in patients on CPAP versus control [32]. Furthermore, in a randomized control trial of 18 patients with OSA who had greater than 10 ventricular premature beats during sleep, Ryan et al. reported a 58% significant reduction in premature beats during sleep in patients treated with CPAP [33–37].

## Conclusion

In this era of pervasive cardiovascular disease, it is imperative to identify and treat potentially modifiable CVD risk factors. Obstructive sleep apnea has revealed itself as a leading, though largely treatable, cause of several CVD entities. The resulting autonomic, inflammatory, hemodynamic and metabolic disturbances of the disease have all been shown to contribute to the pathogenesis and perpetuation of hypertension, type 2 DM, coronary artery disease, heart failure and cardiac arrhythmias. Apart from CAD, treatment of OSA has proven effective in reducing the incidence and prevalence of these cardiovascular disorders. Given the growing evidence regarding the effectiveness of OSA treatment, including CPAP therapy, increased public awareness and resource allocation should be made to enhance early detection and treatment of OSA, a largely modifiable CVD risk factor.
